# Supplementary material for: Unveiling the Bioactive Potential of the Invasive Jellyfish Phyllorhiza punctata Through Integrative Transcriptomic and Proteomic Analyses
Source: Biomolecules. 2025 Aug 4;15(8):1121. doi: 10.3390/biom15081121 (PMC12383608; doi:10.3390/biom15081121)
Supplement: Supplementary file 1 [file biomolecules-15-01121-s001.zip › Suplementary Material/FileS3/PEPFOLD4/XP_065061351_5256/clusters.html]

 PEP-FOLD clusters


Download archive of all models  here   

|  |  |  |
| --- | --- | --- |
| # | file | sOPEP |
| # cluster 1 - Size: 1 |  |  |
|  | XP\_065061351\_5256-model1 | -15.4322 |
| # cluster 2 - Size: 10 |  |  |
|  | XP\_065061351\_5256-model2 | -13.6801 |
|  | XP\_065061351\_5256-model2.1 | -9.28346 |
|  | XP\_065061351\_5256-model2.2 | -9.17301 |
|  | XP\_065061351\_5256-model2.3 | -8.36837 |
|  | XP\_065061351\_5256-model2.4 | -8.29476 |
|  | XP\_065061351\_5256-model2.5 | -8.04175 |
|  | XP\_065061351\_5256-model2.6 | -8.01362 |
|  | XP\_065061351\_5256-model2.7 | -7.13109 |
|  | XP\_065061351\_5256-model2.8 | -7.01802 |
|  | XP\_065061351\_5256-model2.9 | -6.61206 |
| # cluster 3 - Size: 11 |  |  |
|  | XP\_065061351\_5256-model3 | -11.2728 |
|  | XP\_065061351\_5256-model3.1 | -9.70389 |
|  | XP\_065061351\_5256-model3.2 | -9.41028 |
|  | XP\_065061351\_5256-model3.3 | -9.22707 |
|  | XP\_065061351\_5256-model3.4 | -8.97377 |
|  | XP\_065061351\_5256-model3.5 | -8.27215 |
|  | XP\_065061351\_5256-model3.6 | -8.07671 |
|  | XP\_065061351\_5256-model3.7 | -7.62437 |
|  | XP\_065061351\_5256-model3.8 | -7.57546 |
|  | XP\_065061351\_5256-model3.9 | -7.44725 |
|  | XP\_065061351\_5256-model3.10 | -7.02448 |
| # cluster 4 - Size: 2 |  |  |
|  | XP\_065061351\_5256-model4 | -11.2097 |
|  | XP\_065061351\_5256-model4.1 | -8.66891 |
| # cluster 5 - Size: 24 |  |  |
|  | XP\_065061351\_5256-model5 | -10.2718 |
|  | XP\_065061351\_5256-model5.1 | -9.94188 |
|  | XP\_065061351\_5256-model5.2 | -9.85106 |
|  | XP\_065061351\_5256-model5.3 | -9.74701 |
|  | XP\_065061351\_5256-model5.4 | -9.55592 |
|  | XP\_065061351\_5256-model5.5 | -9.43235 |
|  | XP\_065061351\_5256-model5.6 | -9.36286 |
|  | XP\_065061351\_5256-model5.7 | -9.22096 |
|  | XP\_065061351\_5256-model5.8 | -9.12768 |
|  | XP\_065061351\_5256-model5.9 | -9.06609 |
|  | XP\_065061351\_5256-model5.10 | -9.05896 |
|  | XP\_065061351\_5256-model5.11 | -8.88188 |
|  | XP\_065061351\_5256-model5.12 | -8.7559 |
|  | XP\_065061351\_5256-model5.13 | -8.73846 |
|  | XP\_065061351\_5256-model5.14 | -7.89486 |
|  | XP\_065061351\_5256-model5.15 | -7.84516 |
|  | XP\_065061351\_5256-model5.16 | -7.79195 |
|  | XP\_065061351\_5256-model5.17 | -7.71299 |
|  | XP\_065061351\_5256-model5.18 | -7.67118 |
|  | XP\_065061351\_5256-model5.19 | -7.61277 |
|  | XP\_065061351\_5256-model5.20 | -7.41036 |
|  | XP\_065061351\_5256-model5.21 | -6.98218 |
|  | XP\_065061351\_5256-model5.22 | -6.62538 |
|  | XP\_065061351\_5256-model5.23 | -6.46314 |
| # cluster 6 - Size: 2 |  |  |
|  | XP\_065061351\_5256-model6 | -10.2346 |
|  | XP\_065061351\_5256-model6.1 | -7.95853 |
| # cluster 7 - Size: 2 |  |  |
|  | XP\_065061351\_5256-model7 | -9.70006 |
|  | XP\_065061351\_5256-model7.1 | -8.2649 |
| # cluster 8 - Size: 12 |  |  |
|  | XP\_065061351\_5256-model8 | -9.64616 |
|  | XP\_065061351\_5256-model8.1 | -8.4146 |
|  | XP\_065061351\_5256-model8.2 | -8.38733 |
|  | XP\_065061351\_5256-model8.3 | -7.86266 |
|  | XP\_065061351\_5256-model8.4 | -7.7658 |
|  | XP\_065061351\_5256-model8.5 | -7.31938 |
|  | XP\_065061351\_5256-model8.6 | -6.96901 |
|  | XP\_065061351\_5256-model8.7 | -6.84596 |
|  | XP\_065061351\_5256-model8.8 | -6.67137 |
|  | XP\_065061351\_5256-model8.9 | -6.62254 |
|  | XP\_065061351\_5256-model8.10 | -6.62149 |
|  | XP\_065061351\_5256-model8.11 | -6.52266 |
| # cluster 9 - Size: 1 |  |  |
|  | XP\_065061351\_5256-model9 | -9.55593 |
| # cluster 10 - Size: 1 |  |  |
|  | XP\_065061351\_5256-model10 | -9.3858 |
| # cluster 11 - Size: 1 |  |  |
|  | XP\_065061351\_5256-model11 | -9.14718 |
| # cluster 12 - Size: 1 |  |  |
|  | XP\_065061351\_5256-model12 | -9.01827 |
| # cluster 13 - Size: 5 |  |  |
|  | XP\_065061351\_5256-model13 | -8.88065 |
|  | XP\_065061351\_5256-model13.1 | -8.44546 |
|  | XP\_065061351\_5256-model13.2 | -7.38434 |
|  | XP\_065061351\_5256-model13.3 | -7.21774 |
|  | XP\_065061351\_5256-model13.4 | -7.06839 |
| # cluster 14 - Size: 3 |  |  |
|  | XP\_065061351\_5256-model14 | -8.83702 |
|  | XP\_065061351\_5256-model14.1 | -7.93641 |
|  | XP\_065061351\_5256-model14.2 | -7.26027 |
| # cluster 15 - Size: 1 |  |  |
|  | XP\_065061351\_5256-model15 | -8.32181 |
| # cluster 16 - Size: 1 |  |  |
|  | XP\_065061351\_5256-model16 | -8.13652 |
| # cluster 17 - Size: 1 |  |  |
|  | XP\_065061351\_5256-model17 | -8.10401 |
| # cluster 18 - Size: 1 |  |  |
|  | XP\_065061351\_5256-model18 | -8.02241 |
| # cluster 19 - Size: 1 |  |  |
|  | XP\_065061351\_5256-model19 | -7.82578 |
| # cluster 20 - Size: 1 |  |  |
|  | XP\_065061351\_5256-model20 | -7.65252 |
| # cluster 21 - Size: 1 |  |  |
|  | XP\_065061351\_5256-model21 | -7.59196 |
| # cluster 22 - Size: 2 |  |  |
|  | XP\_065061351\_5256-model22 | -7.48519 |
|  | XP\_065061351\_5256-model22.1 | -7.21283 |
| # cluster 23 - Size: 2 |  |  |
|  | XP\_065061351\_5256-model23 | -7.33634 |
|  | XP\_065061351\_5256-model23.1 | -7.16349 |
| # cluster 24 - Size: 4 |  |  |
|  | XP\_065061351\_5256-model24 | -7.31558 |
|  | XP\_065061351\_5256-model24.1 | -6.91818 |
|  | XP\_065061351\_5256-model24.2 | -6.81519 |
|  | XP\_065061351\_5256-model24.3 | -6.43138 |
| # cluster 25 - Size: 2 |  |  |
|  | XP\_065061351\_5256-model25 | -7.19341 |
|  | XP\_065061351\_5256-model25.1 | -6.62237 |
| # cluster 26 - Size: 2 |  |  |
|  | XP\_065061351\_5256-model26 | -7.18976 |
|  | XP\_065061351\_5256-model26.1 | -7.09997 |
| # cluster 27 - Size: 1 |  |  |
|  | XP\_065061351\_5256-model27 | -7.11487 |
| # cluster 28 - Size: 1 |  |  |
|  | XP\_065061351\_5256-model28 | -6.92961 |
| # cluster 29 - Size: 1 |  |  |
|  | XP\_065061351\_5256-model29 | -6.86777 |
| # cluster 30 - Size: 1 |  |  |
|  | XP\_065061351\_5256-model30 | -6.85592 |
| # cluster 31 - Size: 1 |  |  |
|  | XP\_065061351\_5256-model31 | -6.82049 |
|  |  |  |
